# A SNP involved in alternative splicing of *ABCB1* is associated with clopidogrel resistance in coronary heart disease in Chinese population

**DOI:** 10.18632/aging.104177

**Published:** 2020-11-20

**Authors:** Shasha Zhang, Jing Wang, Anan Zhang, Xiaowei Zhang, Tao You, Dingxiong Xie, Wenke Yang, Yuhong Chen, Xuetian Zhang, Cuixia Di, Xiaodong Xie

**Affiliations:** 1 School of Basic Medical Sciences, Lanzhou University, Lanzhou 730000, China; 2 Gansu Provincial Maternity and Childcare Hospital, Lanzhou 730050, China; 3 Bio-Medical Research Center, Institute of Modern Physics, Chinese Academy of Sciences, Lanzhou 730000, China; 4 The Second Hospital of Lanzhou University, Lanzhou 730000, China; 5 Department of Cardiology, The Second Hospital of Lanzhou University, Lanzhou 730000, China; 6 Department of Cardiac Surgery, Gansu Provincial Hospital, Lanzhou 730000, China; 7 Congenital Heart Disease Diagnosis and Treatment Gansu Province International Science and Technology Cooperation Base, Lanzhou 730000, China; 8 Gansu Cardiovascular Institute, Lanzhou 730050, China

**Keywords:** ABCB1, single nucleotide polymorphism, clopidogrel resistance, alternative splicing, silico analysis

## Abstract

Although many scientists are studying the association between genetic polymorphism of *ABCB1* and CR in patients, the molecular mechanism has not been further studied in patients with CHD. This study investigated the relationship between SNP of the *ABCB1* gene in patients with CHD and CR, and whether the polymorphism of the *ABCB1* gene affects the AS of the gene. 741 patients were enrolled in the study, 316 CR cases and 425 NCR cases. The correlation between CR risk and clinical-pathological characteristics were studied. Additionally, the five SNPs were analysed by PCR and Mass Array genotyping methods. Furthermore, silicon analysis was used to predict whether the polymorphism affects the process of AS. Results showed that there was a significant correlation between rs1045642 polymorphism and CR in genotyping and allele analysis. The rs1045642 polymorphism of the *ABCB1* gene of CHD patients carrying the A allele are more likely to develop CR. Silicon analysis showed that rs1045642 generated a new ESE sequence which might affect AS of *ABCB1* gene. We hypothesize that the mechanism of CR might be caused by a change in the AS caused by the polymorphism of the gene. Thus, this work provides guidance for the clinical use of clopidogrel.

## INTRODUCTION

Coronary heart disease (CHD) is usually caused by the lipid accumulation in the walls of the heart arteries [1, 2]. CHD is a high-risk cardiovascular disease in the population and the main cause of death in patients with cardiovascular disease [3, 4]. Percutaneous coronary intervention (PCI) is a treatment for CHD, which might lead to stent thrombosis. Therefore, antithrombotic therapy plays a crucial role in the treatment of CHD [5]. Clopidogrel is mainly prescribed for antiplatelet therapy [6] and ultimately converted into active clopidogrel metabolites in the liver [7]. Although clopidogrel is the cornerstone of antiplatelet therapy, some patients will develop into clopidogrel resistance (CR). Genetic polymorphism is one of the main factors affecting the CR of individuals. Therefore, we can use genetic testing to guide the clinical individualization of CR patients [8–10].

At present, there are many genes in clopidogrel pharmacogenomics research, among which *CYP2C19* and ATP binding cassette subfamily B member 1 (*ABCB1*) genes are particularly concerned. We have selected the *ABCB1* gene for this experimental study of CR and polymorphisms. The p-glycoprotein (p-gp) encoded by the *ABCB 1/MDR 1* (multidrug resistance-1) gene, physiologically, which can transport hydrophobic and hydrophilic compounds through the placenta, intestine and other parts, and protect the blood-brain barrier [11]. In addition, steroid hormones, immunosuppressants and antimetabolites or antibiotics can be discharged, as well as metabolites to protect cells from cytotoxic substances [12]. It causes a variety of drugs to be excreted outside the cancer cells, leading to chemotherapy resistance of tumour [13]. *ABCB1* gene is a highly polymorphic gene with multiple single nucleotide polymorphisms (SNPs). Many studies in recent years had shown that genetic polymorphism affects the process of alternative splicing (AS) and leads to the development and progression of many diseases in humans, such as thalassemia, Alzheimer's disease, male infertility, retinitis pigmentosa and cancer [14–24]. More than 95% of protein coding genes occurs AS that increases the diversity of the human genome [25]. The complex process of AS is affected by many elements. Not only the branch point, the sequence of 3′ and 5′ splice sites but also cis-elements and trans-acting elements are required. Mutations or polymorphisms in cis-regulatory factors might affect mechanical damage of the exon splicing complexes and cause many diseases [16].

Although a large number of scientists around the world are studying the association between genetic polymorphism of *ABCB1* and CR, the results between the genetic polymorphisms and the risk of clopidogrel are still controversial. Diverse results of the above studies might be caused by differences in sample selection requirements, geographical distribution and ethnic differences, sample size, and different genotyping methods [26, 27]. Recently, mutations at specific sites of the gene have caused certain disease and affected the process of the gene's AS [23, 28–31]. As far as we know, the relationship between gene polymorphism and AS in Chinese population with CR risk has not been studied. Therefore, our present study aims to investigate the molecular mechanism of CR caused by the polymorphism of the gene. Our work might provide theoretical guidance for the clinical individualization of clopidogrel in Chinese population with CHD.

## RESULTS

### Distributions of selected variables in cases and controls

The demographic information and clinical characteristics of the CR group and NCR groups were showed in Table 1. The relationship between age and sex in the CR group and NCR group has not been found, indicating that frequency matching of age and gender was appropriate. The mean platelet volume (MPV) was different between the CR group and the NCR group (*P*=0.036). At the same time, the rest of the variables were not found that significant correlation between CR group and NCR group.

**Table 1:** Distributions of selected variables in CHD cases and controls.

**Characteristics**		**NCR**	**CR**	***P***
Total		425	316	
Sex	M (%)	233 (55.1)	190 (44.9)	0.149
	F (%)	192 (60.4)	126 (39.6)	
History of smoking	Y (%)	90 (59.6)	61 (40.4)	0.531
	N (%)	335 (56.8)	255 (43.2)	
Drinking history	Y (%)	34 (66.7)	17(33.3)	0.163
	N (%)	391 (56.7)	299 (43.3)	
History of diabetes	Y (%)	35 (57.4)	26 (42.6)	0.997
	N (%)	390 (57.4)	290 (42.6)	
History of hypertension	Y (%)	114 (56.2)	89 (43.8)	0.686
	N (%)	311 (57.8)	227 (42.2)	
Combined with APC	Y (%)	354 (56.1)	277 (43.9)	0.098
	N (%)	71 (64.5)	39 (35.5)	
Age		63.50±10.68	64.03±9.28	0.479
BMI/kg/m^2^		24.27±3.43	24.16±3.54	0.661
Systolic pressure/mmHg		122.52±16.18	124.38±14.84	0.108
Diastolic pressure/mmHg		74.78±10.20	75.26±9.21	0.509
Glycated hemoglobin/%		5.48±0.71	5.41±0.52	0.151
Triglyceride/mmol/L		1.26±0.62	1.23±0.61	0.561
Total cholesterol/mmol/L		4.37±0.93	4.45±0.96	0.241
HDL-C/mmol/L		1.34±0.26	1.35±0.25	0.850
LDL-C/mmol/L		2.93±0.64	2.98±0.64	0.330
C-reactive protein/mg/L		2.20±3.97	2.54±5.31	0.315
PLT/10^9^		215.42±67.11	219.00±63.35	0.461
MPV/fL		12.55±1.73	12.28±1.68	0.036*
PCT/%		0.23±0.07	0.23±0.07	0.831
PDW/%		17.99±3.78	18.03±3.88	0.900

Abbreviation: CR, Clopidogrel resistance; M, Male; F, Female; Y, Yes; N, No; PLT, Platelet count; MPV, Mean platelet volume; PCT, Plateletocrit; PWD, Platelet volume distribution width.

*: *P* value <0.05 was considered as significant.

### HWE equilibrium test

Sequenom MassArray typing was performed on the above five polymorphisms. We calculated the specific genotype frequencies of the five SNPs and ensured that each site was in HWE equilibrium (Table 2).

**Table 2:** Hardy-Weinberg equilibrium test of five SNPs.

**SNP**	**Frequency *n* (%)**		***P*(NCR)**	***P*(CR)**
	**NCR**	**CR**		
rs1045642	GG (37.9%)/GA(47.5%)/AA(14.6%)	GG (29.7%)/GA(47.5%)/AA(22.8%)	0.916	0.413
rs4148727	TT (81.6%)/TC(17.9%)/CC(0.5%)	TT (82.6%)/TC(17.1%)/CC(0.3%)	0.315	0.302
rs2032582	CC (28.7%)/CA(51.3%)/AA(20.0%)	CC (30.0%)/CA(47.5%)/AA(22.5%)	0.487	0.422
rs3789243	GG (43.5%)/GA(46.4%)/AA(10.1%)	GG (44.6%)/GA(42.1%)/AA(13.3%)	0.369	0.236
rs1858923	AA (27.8%)/AG(50.8%)/GG(21.4%)	AA (26.3%)/AG(49.4%)/GG(24.3%)	0.671	0.827

### Sequence analysis

**Figure 1:**
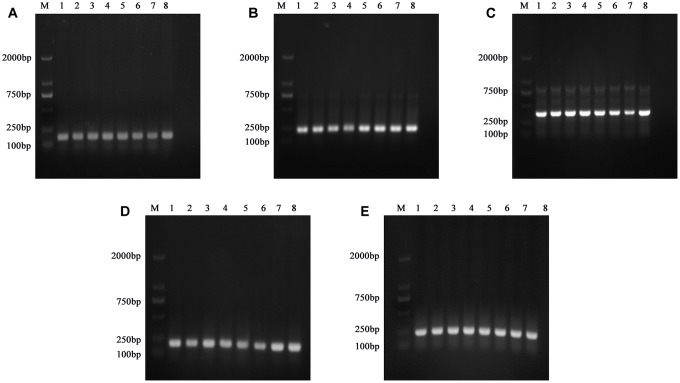
**Electropherograms of PCR product fragments verified by sequence verification of 5 SNPs of partial DNA samples.** (**A**) Electropherogram of PCR product of rs1045642 polymorphism. (**B**) Electropherogram of PCR product of rs4148727 polymorphism. (**C**) Electropherogram of PCR product of rs2032582 polymorphism. (**D**) Electropherogram of PCR product of rs3789243 polymorphism. (**E**) Electropherogram of PCR product of rs1858923 polymorphism.

**Figure 2:**
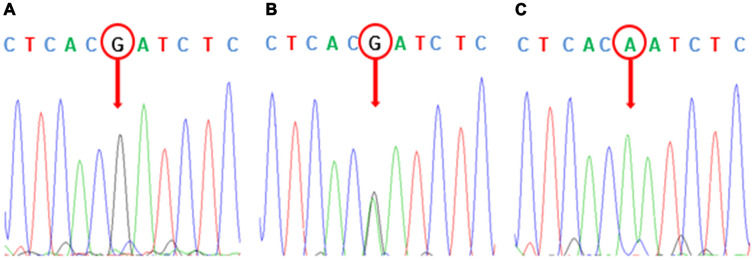
**Sequence verification of rs1045642 polymorphism.** (**A**) GG-wild type homozygous. (**B**) AG-heterozygote. (**C**) AA-SNP homozygous. The arrow in the picture points to the specific bases of the rs1045642 polymorphic sequence.

In order to verify the accuracy of the Sequenom MassArray genotyping method, we randomly selected some samples to verify the sequence of 5 SNPs. The image of agarose gel electrophoresis of the polymerase chain reaction (PCR) amplification products of some samples of DNA samples were shown in Figure 1. The wild homozygous sequence, heterozygous sequence and mutant homozygous sequence of the rs1045642 polymorphic site were listed in Figure 2.

### Statistical analysis of genotyping results of five SNPs

Sequenom MassArray typing technique showed genotypes of 741 participants and we performed a statistical analysis of the genotype results. All clinical data and classification results were entered into SPSS22.0 software. The results were expressed as mean ± standard deviation, and the count data was analysed by chi-square test. Expressed by the number and frequency of distribution, the *P* value reflects the difference between each variable in the CR group and NCR group. The chi-square test of five polymorphisms of *ABCB1* gene was performed to compare the distribution of different genotypes between NCR group and CR group and the results were illustrated in Table 3. Chi-square test showed that the rs1045642 locus of *ABCB1* gene was significantly different between the CR group and the NCR group, while the four polymorphisms (rs4148727, rs2032582, rs3789243, rs1858923) were not significantly different between the groups. The rs1045642 of *ABCB1* gene genotype was distributed in the NCR group at 37.9% (GG, 161), 47.5% (GA, 202), 14.6% (AA, 62), and the genotype distribution frequency in the CR group was 29.7% (GG, 94), 47.5% (GA, 150), 22.8% (AA, 72). In the genotype analysis, we found that rs1045642 AA genotype and GG genotype were significantly different (OR=1.989, 95% CI=1.301-3.040, *P*=0.001); AA genotype and GA + AA genotype were also significantly different (OR=1.440, 95% CI=1.965-1.056, *P*=0.021). In the allele analysis, there was a statistical difference between the allele A and the G allele (OR=1.398, 95% CI=723-1.135, *P*=0.002). The rs1045642 polymorphism of the *ABCB1* gene of CHD patients carrying the A allele is more prone to CR.

**Table 3:** Statistical analysis of the association between five polymorphisms of the *ABCB1* gene and the risk of CR.

**SNP**	**Model**	**Genotype**	**NCR**	**CR**	**OR (95% CI)**	***P***
rs1045642	Heterozygous model	GG	161(44.4%)	94(38.5%)		
		GA	202(55.6%)	150(61.5%)	1.272(0.914-1.770)	0.154
	Homozygous model	GG	161(72.2%)	94(56.6%)		
		AA	62(27.8%)	72(43.4%)	1.989(1.301-3.040)	0.001*
	Dominant model	GG	161(37.9%)	94(29.7%)		
		GA+AA	264(62.1%)	222(70.3%)	1.440(1.056-1.965)	0.021*
	Allele genetic model	G	524(61.6%)	338(53.5%)		
		A	326(38.4%)	294(46.5%)	1.398(1.135-1.723)	0.002*
rs4148727	Heterozygous model	TT	347(82.0%)	261(82.9%)		
		TC	76(18%)	54(17.1%)	0.945(0.643-1.387)	0.771
	Homozygous model	TT	347(99.4%)	261(99.6%)		
		CC	2(0.6%)	1(0.4%)	0.665(0.060-7.370)	0.738
	Dominant model	TT	347(81.6%)	261(82.6%)		
		TC+CC	78(18.4%)	55(17.4%)	0.937(0.641-1.372)	0.739
	Allele genetic model	T	770(90.6%)	576(91.1%)		
		C	80(9.4%)	56(8.9%)	0.936(0.645-1.339)	0.716
rs2032582	Heterozygous model	CC	122(35.9%)	95(38.8%)		
		CA	218(64.1%)	150(61.2%)	0.884(0.629-1.241)	0.475
	Homozygous model	CC	122(58.9%)	95(57.2%)		
		AA	85(41.1%)	71(42.8%)	1.073(0.709-1.622)	0.740
	Dominant model	CC	122(28.7%)	95(30.1%)		
		CA+AA	303(71.3%)	221(69.9%)	0.937(0.681-1.289)	0.688
	Allele genetic model	C	462(54.4%)	340(53.8%)		
		A	388(45.6%)	292(46.2%)	1.023(0.832-1.257)	0.832
rs3789243	Heterozygous model	GG	185(48.4%)	141(51.5%)		
		GA	197(51.6%)	133(48.5%)	0.886(0.649-1.208)	0.444
	Homozygous model	GG	185(81.1%)	141(77.0%)		
		AA	43(18.9%)	42(23.0%)	1.282(0.794-2.068)	0.309
	Dominant model	GG	185(43.5%)	141(44.6%)		
		GA+AA	240(56.5%)	175(55.4%)	0.957 (0.714-1.283)	0.767
	Allele genetic model	G	567(66.7%)	415(65.7%)		
		A	283(33.3%)	217(34.3 %)	1.048(0.843-1.302)	0.678
rs1858923	Heterozygous model	AA	118(35.3%)	83(34.7%)		
		AG	216(64.7%)	156(65.3%)	0.974(0.688-1.380)	0.882
	Homozygous model	AA	118(56.5%)	83(51.9%)		
		GG	91(43.5%)	77(48.1%)	0.831(0.550-1.257)	0.381
	Dominant model	AA	118(27.8%)	83(26.3%)		
		AG+GG	307(72.2%)	233(73.7%)	0.927(0.667-1.287)	0.650
	Allele genetic model	A	452(53.2%)	322(50.9%)		
		G	398(46.8%)	310(49.1%)	0.915(0.744-1.124)	0.396

*: *P* value <0.05 was considered as significant.

### LD and haplotype analysis

**Figure 3:**
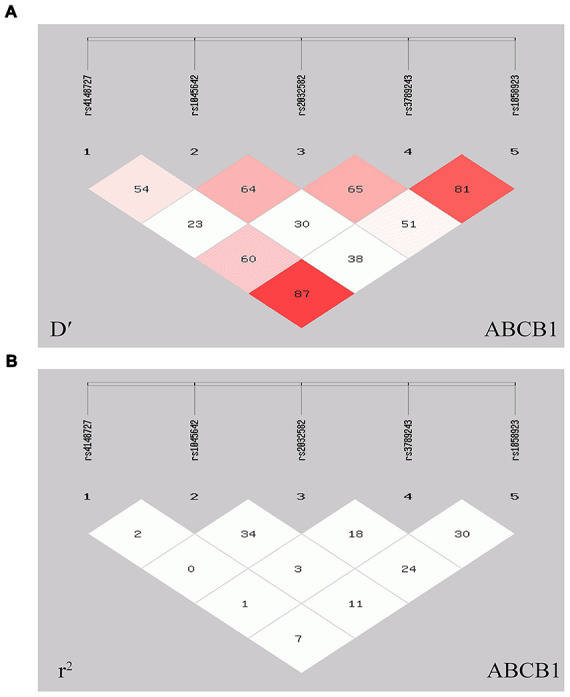
**Linkage disequilibrium plot and *r*^2^ values for five polymorphisms of the *ABCB1* gene.** (**A**) D′ plot of five polymorphic linkage disequilibrium. (**B**) *r*^2^ plot of five polymorphic linkage disequilibrium. Linkage disequilibrium was detected in the rs3789243 and rs1858923 polymorphisms ((*r*^2^ = 0.310, D′ = 0.815), rs1858923 and rs4148727 polymorphisms (*r*^2^ = 0.070, D′ = 0.872).

SHEsis software was used to analyze the LD of five polymorphisms of *ABCB1*. In the Figure 3 and Table 4, LD was detected in the rs3789243 and rs1858923 polymorphisms (*r*^2^=0.310, D′=0.815), rs1858923 and rs4148727 polymorphisms (*r*^2^=0.070, D′=0.872). Meanwhile, haplotype analysis was performed in CR and NCR groups. In this study, the CR group and NCR group with haplotype estimated frequency below 3% were excluded from further analysis. We found that TGAGG haplotypes (rs4148727, rs1045642, rs2032582, rs3789243, rs1858923.) can appreciably lower the risk of CR (OR=0.663, 95% CI=0.447-0.983, *P*=0.040), and other haplotypes were not significantly different between CR group and NCR groups (Table 5).

**Table 4:** SHEsis software analyzes the LD relationship of five polymorphisms of *ABCB1*, and uses D′ and *r*^2^ to measure the LD relationship between the five SNPs.

**D′**	**SNP**	**rs1045642**	**rs2032582**	**rs3789243**	**rs1858923**
	rs4148727	0.546	0.237	0.608	0.872
	rs1045642	-	0.642	0.303	0.385
	rs2032582	-	-	0.656	0.516
	rs3789243	-	-	-	0.815
***r*^2^**	**SNP**	**rs1045642**	**rs2032582**	**rs3789243**	**rs1858923**
	rs4148727	0.022	0.022	0.019	0.070
	rs1045642	-	0.349	0.034	0.116
	rs2032582	-	-	0.186	0.247
	rs3789243	-	-	-	0.310

Abbreviation: LD, linkage disequilibrium.

**Table 5:** Results of haplotype analysis of five *ABCB1* SNPs in CR and control groups.

Haplotype*	**Frequency *n* (%)**		***P***	**OR (95%CI)**
	**CR**	**NCR**		
C G C G A	26.78(0.042)	42.98(0.051)	0.456	0.828 (0.505~1.360)
T A A A G	20.89(0.033)	14.85(0.017)	0.053	1.924 (0.980~3.776)
T A A G A	29.78(0.047)	36.67(0.043)	0.718	1.096 (0.667~1.800)
T A A G G	165.44(0.262)	185.45(0.218)	0.05	1.282 (1.002~1.640)
T A C A A	33.67(0.053)	27.79(0.033)	0.05	1.667 (0.996~2.789)
T G A G G	40.77(0.065)	79.64(0.094)	0.04*	0.663 (0.447~0.983)
T G C A A	143.43(0.227)	208.64(0.245)	0.388	0.896 (0.699~1.149)
T G C G A	32.44(0.051	59.72(0.070)	0.132	0.713 (0.458~1.109)
T G C G G	63.03(0.100)	91.22(0.107)	0.626	0.919 (0.653~1.293)

Abbreviation: CR, Clopidogrel resistance; OR, Odds ratio; CI, Confidence interval.

The order of SNPs in haplotypes: rs4148727, rs1045642, rs2032582, rs3789243, rs1858923.

*: *P* value <0.05 was considered as significant.

### Silico analysis of rs1045642 polymorphism

**Figure 4:**
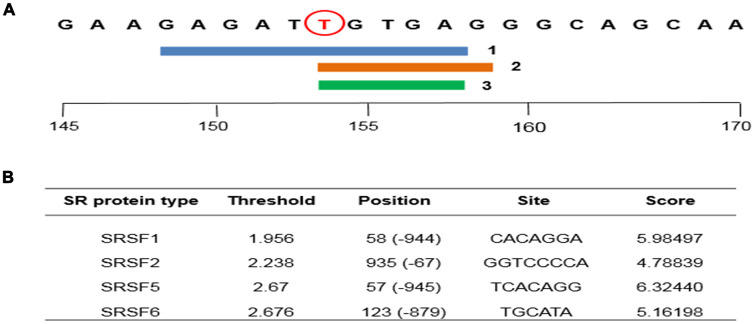
**Human Splicing Finder version 3.1 (HSF) and ESE finder 3.0 bioinformatics analysis softwares are used to predict the effect the mutation of the rs1045642 SNP of the *ABCB1* gene on AS.** (**A**) The three algorithms of HSF software predict that polymorphism is an ESE site. (**B**) ESE finder software predicts the binding ability of ESE site to SRps.

Mutations in cis-elements and splice sites can affect gene splicing. These effects were detected by HSF 3.1 and ESE finder 3.0. The HSF 3.1 software was used to predict whether the mutation of the rs1045642 SNP of the *ABCB1* gene has an effect on AS, which predicted that a new ESE sequence was generated by mutation of the rs1045642 SNP of the *ABCB1* gene (Figure 4A). Since the ESE sequence is a cis-acting element of AS, we further used ESE-finder software to predict whether the mutation of the gene would alter the ability to bind the trans-acting factor SR proteins in the AS. The ESE-finder software predicted that the new ESE sequence generated by the mutation will bind to the four SR proteins beyond the binding threshold (Figure 4B). Thus, silicon analysis showed that rs1045642 generated a new ESE sequence which might affect AS of *ABCB1* gene.

## DISCUSSION

*ABCB1*, P-glycoprotein, is an ABC transporter whose main function of ABCB1 is to expel toxic metabolites and xenobiotics from cells [32, 33]. Therefore, the ABCB1 gene has a great development in the mechanism of anticancer drug resistance [34]. Previous studies had reported gene polymorphisms and CR, and the *ABCB1* is one of the hot genes. In our research, we have selected five polymorphisms of rs1045642, rs4148727, rs2032582, rs3789243 and rs1858923 on the *ABCB1* gene to study the relationship between *ABCB1* gene polymorphisms and the risk of CR. Meanwhile, the polymorphism rs1045642 of *ABCB1* gene is more frequently studied in CR. In recent years, there have been different studies between the *ABCB1* gene polymorphism and the occurrence of CR in patients with CHD. Our research showed that four polymorphisms of rs4148727, rs2032582, rs3789243 and rs1858923 were not associated with CR. However, the rs1045642 polymorphism was found to be intensively associated with CR.

Previous studies have shown that the concentration of active clopidogrel metabolites in homozygous 3435TT was significantly lower than that of CT/CC, suggesting that the increased expression of p-glycoprotein associated with 3435TT genotype might mediate the increase of clopidogrel efflux in the intestine [35, 36]. Therefore, it showed that *ABCB1* gene polymorphism was related to the efficacy of clopidogrel in patients. We further studied the *ABCB1* gene polymorphism and CR and found that the TT homozygous rs1045642 mutation has an increased risk of CR compared with CT/CC individuals. People with T alleles have an increased risk of ischemic events. Our results were consistent with those of Jessica L Mega et al. [37]. However, the study on CR and rs1045642 polymorphisms by Jia Su et al. found that there was no significant correlation between rs1045642 mutation and CR [38]. The inconsistency of the research results might be related to different sample selection standards, geographical distribution and ethnic differences, sample size and different genotyping methods. Currently, there are no prescribed standard for CR. Flow cytometry vasodilator-stimulated phosphoprotein phosphorylation (Vasp) analysis and verification-Now P2Y12 detection, pfa-100, whole TEG and impedance aggregation metrology (multi-plate analyzer) can all be used detect platelet function and clopidogrel reaction [39]. The method of evaluating CR is different, which might have an impact on our further research on CR. In this study, we had used TEG method to measure platelet inhibition rate. The VerifyNow analysis P2Y12 method was used by Jia Su et al. Previous studies had confirmed that allelic variants of the *CYP2C19* gene have ethnic and geographic diversity, but they were not obvious in Asians or mixed races, which indicated that different genetic backgrounds have different frequencies of mutations [40]. In addition, studies had shown that polymorphisms of enzyme genes that metabolize drugs might change the response to drugs and occur at different frequencies in different ethnic groups [41]. This indicates that genetic background might change the effect of mutations on treatment in a specific group. Our samples were collected in Northwest China, and the samples in the study of Jia Su et al. were recruited in East China. Richard C Crist's study concluded that due to the small sample size, many previous studies were insufficient for retrospective drug genetic studies of African Americans [41]. The sample size is too small to detect experimental effect. The difference in sample size might also affect the results of experimental studies. 741 samples were included in our study, and 180 samples were included in the study of Jia Su et al., which was far less than the sample size of our experiment. At present, there are many methods for genotyping, including PCR-RFLP (polymerase chain reaction-restricted fragment length polymorphism) method, Taq-Man probe, ARMS (amplification difficult mutation system), genechip, Sanger sequencing, Mass spectrometry, etc. Different genotyping methods have their advantages and shortcomings. The typing method used in our experiment was Sequenom MassArray, and the PCR-RFLP genotyping method was used by Jia Su et al. The advantages of the Sequenom MassArray method include low cost, no need for special fluorescent primers, only a pair of PCR primers and amplification primers, convenient detection, high sensitivity, and high data accuracy. However, the PCR-RFLP method requires a specific restriction site for genotyping SNPs, and it is impossible to accurately identify false positives. Therefore, our typing method is better than the typing method used by Jia Su et al.

Compared with previous CR-related studies, our study not only analysed five polymorphisms of the gene, but also used bioinformatic software to predict the relationship between rs1045642 polymorphism and AS. More than 50% of all mutations leading to genetic diseases result in abnormal splicing [42]. Different forms of cis-regulatory elements include exon splicing enhancer (ESE), exon splicing silencer, intron splicing enhancer and intron splicing silencer sequences. The trans-acting factors are mainly SR proteins protein and heterogeneous nuclear ribonucleoproteins [16]. The combination of cis-regulatory element mutation/polymorphism and trans-acting factor leads to mechanical cleavage of exon splicing complex, which is an important mechanism for AS to participate in the development and progression of various diseases. Preliminary studies indicated that exon polymorphism of *CD44* gene was analysed by ESE finder 3.0 demonstrated that mutation generated a new SR proteins binding site [43]. Therefore, we used HSF 3.1 to predict the rs1045642 polymorphism to generate a new ESE sequence. Furthermore, it was analysed by software (ESE finder 3.0) analysis that the binding ability of ESE sequence to SR proteins exceeded their binding thresholds. The bioinformatics software predicted that the rs1045642 polymorphism is a potential splice site, and the altered binding ability of the ESE sequence and the SR protein formed by the mutation affects the abnormal process of the AS of the *ABCB1* gene. Therefore, we suspect that there is a relationship between the risk of CR in patients with CHD and the rs1045642 polymorphism affecting the AS process of *ABCB1* gene.

**Figure 5:**
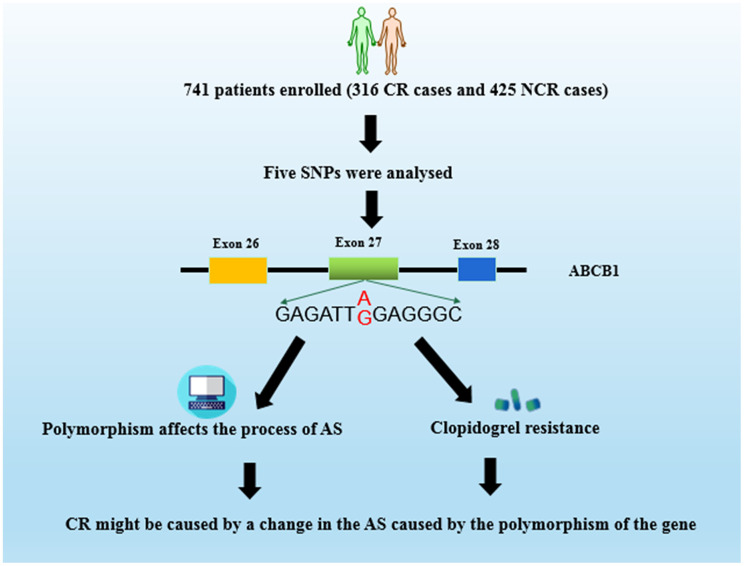
**Schematic representation of mechanism of genetic polymorphism of *ABCB1* and clopidogrel resistance (CR) in patients.**

In our research, there was a significant relationship between the risk of CR in CHD patients and the different genotypes carried by individuals in China. Meanwhile, bioinformatics software has predicted that the *ABCB1* gene rs1045642 polymorphism affects the process of AS and might have a certain relationship with the risk of CR in CHD patients (Figure 5). This study not only provides theoretical support for the individualized administration of clopidogrel after PCI in clinical CHD patients, but also offers a new basis for CR risk in CHD patients and the change of AS process caused by *ABCB1* gene polymorphism.

There is still controversial about the study between CR and polymorphism in patients with CHD. Previous studies on CR only performed polymorphic genotyping by collecting basic clinical information, test results, and patient blood samples, and then statistical analysis to obtain genotypic analysis and CR. However, the mechanism of clopidogrel-related drug resistance has not been further studied. In the current study, we not only did the predecessor research, but also predicted the SNP of clopidogrel-related genes through bioinformatic software. It was predicted that rs1045642 SNP site will potentially affect the AS of the *ABCB1* gene. Therefore, we hypothesize that the mechanism of CR might be caused by a change in the AS caused by the polymorphism of the gene. Our research not only provides theoretical guidance for the clinical individualization of clopidogrel in patients with CHD, but also provides new idea for the study of the mechanism of CR. Meanwhile, specific experiments are needed to support our findings.

## MATERIALS AND METHODS

### Study population

The subjects of this case-control study were 741 patients with CHD or taking clopidogrel hydrogen sulfate after PCI in the Second Hospital of Lanzhou University and Gansu Provincial People's Hospital in Lanzhou City, Gansu Province, China. Briefly, there were 316 patients in the CR group and 425 patients in the NCR group. The exclusion criteria of this study were as follows: (1) the subjects were under 18 years old; (2) patients with a history of clopidogrel allergy; (3) severe liver and kidney insufficiency; (4) peptic ulcer; (6) major surgery; (7) cerebrovascular accident or recent bleeding history. Clinical data were collected from the electronic records, including demographic characteristics, history of smoking, history of drinking, history of hypertension, history of diabetes and previous history of major medical conditions. Laboratory tests, including triglycerides, total cholesterol, high-density lipoprotein cholesterol, low-density lipoprotein cholesterol, glycosylated hemoglobin, high-sensitivity C-reactive protein and platelet count.and other biochemical variables, were performed after admission. This study was approved by the Ethics Committee of Lanzhou University School of Basic Medicine.

### Thromboelastography test

The platelet inhibition rate was detected by thromboelastography (TEG) method according to the following steps: (1) 1 ml of citrated blood was mixed with kaolin by inversion and 340 μl of the activated blood was added to the 20 μl of calcium chloride; (2) 360 μl heparin anticoagulated blood was added to 10 μl batroxobin mixture; (3) 10 μl batroxobin and 10 μl ADP mixture were added to 360 μl heparin blood; (4) the CFMSLEPU-8800 instrument was used to detect the blood clot elasticity map. The test was performed using the CFMSLEPU-8800 Analyzer System. At present, there is no standardized baseline for CR. In this study, ADP inhibition rate <20% was classified as CR, and ADP inhibition rate ≥50% was defined as NCR [44].

### SNPs selection

The *ABCB1* gene is located on human chromosome 7 at q21 [45]. This study referred to NCBI (http://www.ncbi.nlm.nih.gov/ncbisearch), Ensembl (http://asia.ensembl.org/index.html/), the HapMap database of Han Chinese in Beijing, China (http://hapmap.ncbi.nlm.nih.gov) Databases and published articles related to CR when selecting the SNPs of the *ABCB1* gene [46]. Ultimately, five polymorphisms rs1045642, rs4148727, rs2032582, rs3789243 and rs1858923 were selected in the current study.

### Extraction of genomic DNA

Genomic DNA was extracted from EDTA-treated peripheral blood using the phenol/chloroform method. The extracted DNA was dissolved in 50 μl of 1 × TE buffer and frozen (−80° C) until required. The NanoDrop 2000c spectrophotometer (Thermo Fisher Scientific, Lenexa, KS, USA) was used to detect the concentration and purity of DNA. Samples with DNA concentration exceeding 20 ng/ul were sent to biological company (Bomiao Biotechnology Co., Ltd., Beijing, China) for genotyping.

### PCR amplification

The extracted genomic DNA was amplified by PCR. The PCR primers were designed using Assay design 3.1 software and submitted to Shanghai Invitrogen Biotechnology Co., Ltd. for synthesis. The amplification primers were shown in Table 6. For amplification of the DNA, the thermal cycling involved initial denaturation at 95° C for 5 min, 45 cycles of 95° C for 20 s, 56° C for 30 s, and 72° C for 1 min, and a final extension at 72° C for 7 min.

**Table 6:** Primers for polymerase chain reaction.

**Gene**	**SNP**	**Group**	**Base sequence (5′ → 3′)**	**Products size**
*ABCB1*	rs1045642	Forward primer	ACGTTGGATGTTGCCTATGGAGACAACAGC	
		Reverse primer	ACGTTGGATGAAGGCATGTATGTTGGCCTC	99bp
	rs4148727	Forward primer	ACGTTGGATGCTTCAATGCTTTGGAGCCAT	
		Reverse primer	ACGTTGGATGTAAGTTTGGGTGGAGGAAGG	98bp
	rs2032582	Forward primer	ACGTTGGATGCATATTTAGTTTGACTCACC	
		Reverse primer	ACGTTGGATGTGTTGTCTGGACAAGCACTG	92bp
	rs3789243	Forward primer	ACGTTGGATGATAAGCCCAAGATCCTGTCC	
		Reverse primer	ACGTTGGATGCTGACTGCTTCAGTTCCAAC	107bp
	rs1858923	Forward primer	ACGTTGGATGTCTGACTCCTGTGATAAGGG	
		Reverse primer	ACGTTGGATGTGCTGATTTTCCTCCAGCTC	109bp

### Identification of ABCB1 SNPs by Sequenom MassArray

Extracted DNA samples were sent to a biological company (Bomiao Biotechnology Co., Ltd., Beijing, China) for genotyping. Sequenom MassArray typing technique was used to determine the all individual genotypes. The specific steps of the Sequenom MassArray genotyping method are as follows: (1) The PCR amplification primer sequences and extension primer sequences of genotyping SNPs were designed, and the primer sequences were sent Shanghai Invitrogen Biotechnology Co., Ltd for synthesis; (2) 4 μl PCR master mix and 1 μl template DNA were added to each well of the 384-well plate. The PCR reaction program was 94° C, 5 min; 45 cycles: 94° C, 20 s, 56° C, 30 s, 72° C, 1 min; 72° C, 3 min; 4° C, ∞; (3) The PCR product was treated with shrimp alkaline phosphatase (SAP) to remove free dNTPs in the system, and 2 μl SAP mixture and 5 μl PCR product were added to each well of 384-well plate for reaction. The SAP reaction program was37° C, 20 min; 85° C, 5 min; 4° C, ∞; (4) 2 μl single base mixture and 7 μl SAP-PCR reaction product were added to each well of 384-well plate. The extension reaction program was 90° C, 30 s; 40 cycles: 94° C, 5 s, (52° C, 5 s, 80° C, 5 s) × 5 cycles; 72° C, 3 min; 4° C, ∞; (5) Purification reaction; (6) Chip spotting; (7) Mass spectrometry detection and data output. After the MALDI-TOF mass spectrometer analyzer detects the SpectroCHIP chip, TYPER4.0 software was used to further analyze and process the raw data.

### Sequencing verification

Conclusively, partial PCR products were randomly selected for direct sequencing verification. PRIMER 3.0 software was used for primer design and sent to General Biosystems (Anhui) Co., Ltd. for synthesis. The synthesized primer sequence and PCR product size were shown in Table 7. The PCR reaction program was pre-denaturation 94° C for 3 min, 31 cycles of 94° C for 30 s, 58.6° C for 30 s, and 72° C for 25 s, and a final extension at 72° C for 5 min. In the 1.1% agarose gel pool in 1×TAE electrophoresis solution, the PCR products (5 μl/well) of each sample were electrophoresed at 110 V for 25 min, and the agarose was treated with GoldView I. The target bands were detected on the Tanon 1600 gel imaging system (Tanon Science and Technology Co., Ltd.), and the amplified products were sent to General Biosystems (Anhui) Co., Ltd. for sequencing verification. The sequencing results were analysed by SeqManII software (DNASTAR, Madison, WI, USA).

**Table 7:** Primers for polymerase chain reaction.

**Gene**	**SNP**	**Group**	**Base sequence (5′ → 3′)**	**Products size**
*ABCB1*	rs1045642	Forward primer	GCTGAGAACATTGCCTATGG	
		Reverse primer	GGAAGTGTGGCCAGATGCTTGT	259bp
	rs4148727	Forward primer	CATTCTGCCTATTCTGGCTA	
		Reverse primer	GGATAAGTTTGGGTGGAGGA	232bp
	rs2032582	Forward primer	GCTATAGGTTCCAGGCTTGC	
		Reverse primer	GTCCAAGAACTGGCTTTGCT	390bp
	rs3789243	Forward primer	CCATTCCTTGAGGACTTTGG	
		Reverse primer	CCCAGACAATAAGCCCAAGA	202bp
	rs1858923	Forward primer	TGTCTTGATTTCGCCATTGA	
		Reverse primer	CCTGTGATAAGGGGTTGAGG	246bp

### Statistical analysis

SPSS 22.0 statistical software (IBM Corp. Armonk, NY, USA) was used for data analysis. For the continuous variables, analysis of variance techniques or Kruskal-Wallis tests were used and expressed as the means ± SD. Categorical variables are summarised by frequency and percentage. Associations between SNPs and CR in CHD were assessed using the Chi-square test. Hardy-Weinberg equilibrium (HWE), linkage disequilibrium (LD) and haplotype analysis tests were evaluated using SHEsis software (http://analysis.bio-x.cn/myAnalysis.php) in case and control groups. Odds ratio (OR) with 95% Confidence intervals (CI) were calculated to estimate the strength of relationship between individual genotypes of five polymorphisms and CR. *P* value < 0.05 for the difference was significant.

### Silico analysis

In order to determine whether rs1045642 affects the splicing efficiency, computer analysis was performed using HSF 3.1 and ESE finder 3.0 bioinformatics analysis software [42, 43, 45]. HSF 3.1 detects receptor (3'ss) and donor (5'ss) positions by a position weight matrix. ESE finder 3.0 software was used to estimate ESE sequences that change due to mutations. The default threshold was used to determine the locus responsible for the four Serine/arginine-rich (SR) proteins, including AS factor/splicing factor 2 (ASF / SF2), SR splicing factor 5 (SRp40), SR splicing factor 3 (SC35), SR splicing factor 6 (SRp55). The default thresholds for HSF were 1.956, 2.67, 2.383, and 2.6676, respectively. Only sequences with scores above or equal to the threshold were selected.

### Availability of data and materials

The datasets generated and analyzed during the current study are available from the corresponding author on reasonable request.

## References

[1] Wardoku R, Blair C, Demmer R, Prizment A. Association between physical inactivity and health-related quality of life in adults with coronary heart disease. Maturitas. 2019; 128:36–42. 10.1016/j.maturitas.2019.07.005 31561820 PMC7261413

[2] von Känel R, Carney RM, Zhao S, Whooley MA. Heart rate variability and biomarkers of systemic inflammation in patients with stable coronary heart disease: findings from the heart and soul study. Clin Res Cardiol. 2011; 100:241–47. 10.1007/s00392-010-0236-5 20857123 PMC3207966

[3] Jin X, Pan B, Wu H, Wu B, Li Y, Wang X, Liu G, Dang X, Xu D. The efficacy and safety of shenzhu guanxin recipe granules for the treatment of patients with coronary artery disease: protocol for a double-blind, randomized controlled trial. Trials. 2019; 20:520. 10.1186/s13063-019-3629-4 31429810 PMC6701014

[4] Mikrani R, Li C, Naveed M, Li C, Baig MM, Zhang Q, Wang Y, Peng J, Zhao L, Zhou X. Pharmacokinetic advantage of ASD device promote drug absorption through the epicardium. Pharm Res. 2020; 37:173. 10.1007/s11095-020-02898-6 32839887

[5] Moulias A, Alexopoulos D. Long-term outcome of percutaneous coronary intervention: the significance of native coronary artery disease progression. Clin Cardiol. 2011; 34:588–92. 10.1002/clc.20929 21932326 PMC6652501

[6] Patti G, Micieli G, Cimminiello C, Bolognese L. The role of clopidogrel in 2020: a reappraisal. Cardiovasc Ther. 2020; 2020:8703627. 10.1155/2020/8703627 32284734 PMC7140149

[7] Fox KA, Chelliah R. Clopidogrel: an updated and comprehensive review. Expert Opin Drug Metab Toxicol. 2007; 3:621–31. 10.1517/17425225.3.4.621 17696811

[8] Zhang X, Wang Y. [Status quo and countermeasure of clopidogrel resistance predicted by gene testing]. Zhonghua Yi Xue Yi Chuan Xue Za Zhi. 2019; 36:649–53. 10.3760/cma.j.issn.1003-9406.2019.06.030 31055828

[9] Pereira NL, So D, Bae JH, Chavez I, Jeong MH, Kim SW, Madan M, Graham J, O’Cochlain F, Pauley N, Lennon RJ, Bailey K, Hasan A, et al. International survey of patients undergoing percutaneous coronary intervention and their attitudes toward pharmacogenetic testing. Pharmacogenet Genomics. 2019; 29:76–83. 10.1097/FPC.0000000000000368 30724853 PMC6476684

[10] Su J, Zheng N, Li Z, Huangfu N, Mei L, Xu X, Zhang L, Chen X. Association of GCK gene DNA methylation with the risk of clopidogrel resistance in acute coronary syndrome patients. J Clin Lab Anal. 2020; 34:e23040. 10.1002/jcla.23040 31605429 PMC7031555

[11] Martin-Orozco E, Sanchez-Fernandez A, Ortiz-Parra I, Ayala-San Nicolas M. Wnt signaling in tumors: the way to evade drugs and immunity. Front Immunol. 2019; 10:2854. 10.3389/fimmu.2019.02854 31921125 PMC6934036

[12] Cario E. P-glycoprotein multidrug transporter in inflammatory bowel diseases: more questions than answers. World J Gastroenterol. 2017; 23:1513–20. 10.3748/wjg.v23.i9.1513 28321153 PMC5340804

[13] Zhang M, Chen XY, Dong XD, Wang JQ, Feng W, Teng QX, Cui Q, Li J, Li XQ, Chen ZS. NVP-CGM097, an HDM2 inhibitor, antagonizes ATP-binding cassette subfamily B member 1-mediated drug resistance. Front Oncol. 2020; 10:1219. 10.3389/fonc.2020.01219 32793491 PMC7390918

[14] Mijajlovic MD, Shulga O, Bloch S, Covickovic-Sternic N, Aleksic V, Bornstein NM. Clinical consequences of aspirin and clopidogrel resistance: an overview. Acta Neurol Scand. 2013; 128:213–19. 10.1111/ane.12111 23432706

[15] Huang BY, Zeng Y, Li YJ, Huang XJ, Hu N, Yao N, Chen MF, Yang ZG, Chen ZS, Zhang DM, Zeng CQ. Uncaria alkaloids reverse ABCB1-mediated cancer multidrug resistance. Int J Oncol. 2017; 51:257–68. 10.3892/ijo.2017.4005 28534954 PMC5467778

[16] Li Y, Yuan Y. Alternative RNA splicing and gastric cancer. Mutat Res. 2017; 773:263–73. 10.1016/j.mrrev.2016.07.011 28927534

[17] Thein SL. The molecular basis of β-thalassemia. Cold Spring Harb Perspect Med. 2013; 3:a011700. 10.1101/cshperspect.a011700 23637309 PMC3633182

[18] Vasquez JB, Simpson JF, Harpole R, Estus S. Alzheimer’s disease genetics and ABCA7 splicing. J Alzheimers Dis. 2017; 59:633–41. 10.3233/JAD-170872 28655137 PMC5890296

[19] Rafaee A, Mohseni Meybodi A, Yaghmaei P, Hosseini SH, Sabbaghian M. Single-nucleotide polymorphism c.474G>A in the SEPT12 gene is a predisposing factor in male infertility. Mol Reprod Dev. 2020; 87:251–59. 10.1002/mrd.23310 31880374

[20] Shankar SP, Birch DG, Ruiz RS, Hughbanks-Wheaton DK, Sullivan LS, Bowne SJ, Stone EM, Daiger SP. Founder effect of a c.828+3A>T splice site mutation in peripherin 2 (PRPH2) causing autosomal dominant retinal dystrophies. JAMA Ophthalmol. 2015; 133:511–17. 10.1001/jamaophthalmol.2014.6115 25675413 PMC4449732

[21] Littink KW, Pott JW, Collin RW, Kroes HY, Verheij JB, Blokland EA, de Castro Miró M, Hoyng CB, Klaver CC, Koenekoop RK, Rohrschneider K, Cremers FP, van den Born LI, den Hollander AI. A novel nonsense mutation in CEP290 induces exon skipping and leads to a relatively mild retinal phenotype. Invest Ophthalmol Vis Sci. 2010; 51:3646–52. 10.1167/iovs.09-5074 20130272

[22] Wang Y, Freedman JA, Liu H, Moorman PG, Hyslop T, George DJ, Lee NH, Patierno SR, Wei Q. Associations between RNA splicing regulatory variants of stemness-related genes and racial disparities in susceptibility to prostate cancer. Int J Cancer. 2017; 141:731–43. 10.1002/ijc.30787 28510291 PMC5512873

[23] Xiao F, Zhang P, Wang Y, Tian Y, James M, Huang CC, Wang L, Wang L. Single-nucleotide polymorphism rs13426236 contributes to an increased prostate cancer risk via regulating MLPH splicing variant 4. Mol Carcinog. 2020; 59:45–55. 10.1002/mc.23127 31659808 PMC7219604

[24] Zhao D, Zhang C, Jiang M, Wang Y, Liang Y, Wang L, Qin K, Rehman FU, Zhang X. Survival-associated alternative splicing signatures in non-small cell lung cancer. Aging (Albany NY). 2020; 12:5878–93. 10.18632/aging.102983 32282333 PMC7185095

[25] Gallo CM, Ho A, Beffert U. ApoER2: functional tuning through splicing. Front Mol Neurosci. 2020; 13:144. 10.3389/fnmol.2020.00144 32848602 PMC7410921

[26] Tsantes AE, Ikonomidis I, Papadakis I, Bonovas S, Gialeraki A, Kottaridi C, Kyriakou E, Kokori S, Douramani P, Kopterides P, Karakitsos P, Lekakis J, Kapsimali V. Impact of the proton pump inhibitors and CYP2C19*2 polymorphism on platelet response to clopidogrel as assessed by four platelet function assays. Thromb Res. 2013; 132:e105–11. 10.1016/j.thromres.2013.06.015 23830212

[27] Akram N, Mustafa G, Hanif AA, Tawwab S, Hussain S, Kaul H, Mohsin S. Cytochrome 2C19 and paraoxonase-1 polymorphisms and clopidogrel resistance in ischemic heart disease patients. Per Med. 2019. [Epub ahead of print]. 10.2217/pme-2018-0030 31591927

[28] Xue LL, Wang F, Xiong LL, Du RL, Zhou HL, Zou Y, Wu MX, Yang MA, Dai J, He MX, Wang TH. A single-nucleotide polymorphism induced alternative splicing in Tacr3 involves in hypoxic-ischemic brain damage. Brain Res Bull. 2020; 154:106–15. 10.1016/j.brainresbull.2019.11.001 31722250

[29] Mascarenhas JB, Tchourbanov AY, Fan H, Danilov SM, Wang T, Garcia JG. Mechanical stress and single nucleotide variants regulate alternative splicing of the MYLK gene. Am J Respir Cell Mol Biol. 2017; 56:29–37. 10.1165/rcmb.2016-0053OC 27529643 PMC5248959

[30] Singh RK, Cooper TA. pre-mRNA splicing in disease and therapeutics. Trends Mol Med. 2012; 18:472–82. 10.1016/j.molmed.2012.06.006 22819011 PMC3411911

[31] Tan W, Wang W, Ma Q. Physiological and pathological function of serine/arginine-rich splicing factor 4 and related diseases. Biomed Res Int. 2018; 2018:3819719. 10.1155/2018/3819719 29789787 PMC5896335

[32] Ofverholm A, Einbeigi Z, Manouchehrpour S, Albertsson P, Skrtic S, Enerbäck C. The ABCB1 3435 T allele does not increase the risk of paclitaxel-induced neurotoxicity. Oncol Lett. 2010; 1:151–54. 10.3892/ol_00000028 22966274 PMC3436394

[33] Huang YW, Lin CY, Tsai HC, Fong YC, Han CK, Huang YL, Wu WT, Cheng SP, Chang HC, Liao KW, Wang SW, Tang CH. Amphiregulin promotes cisplatin chemoresistance by upregulating ABCB1 expression in human chondrosarcoma. Aging (Albany NY). 2020; 12:9475–88. 10.18632/aging.103220 32428872 PMC7288968

[34] Parle-McDermott A, Pangilinan F, O’Brien KK, Mills JL, Magee AM, Troendle J, Sutton M, Scott JM, Kirke PN, Molloy AM, Brody LC. A common variant in MTHFD1L is associated with neural tube defects and mRNA splicing efficiency. Hum Mutat. 2009; 30:1650–56. 10.1002/humu.21109 19777576 PMC2787683

[35] Taubert D, von Beckerath N, Grimberg G, Lazar A, Jung N, Goeser T, Kastrati A, Schömig A, Schömig E. Impact of p-glycoprotein on clopidogrel absorption. Clin Pharmacol Ther. 2006; 80:486–501. 10.1016/j.clpt.2006.07.007 17112805

[36] Stokanovic D, Nikolic VN, Konstantinovic SS, Zvezdanovic JB, Lilic J, Apostolovic SR, Pavlovic M, Zivkovic VS, Jevtovic-Stoimenov T, Jankovic SM. P-glycoprotein polymorphism C3435T is associated with dose-adjusted clopidogrel and 2-oxo-clopidogrel concentration. Pharmacology. 2016; 97:101–06. 10.1159/000442712 26695516

[37] Mega JL, Close SL, Wiviott SD, Shen L, Walker JR, Simon T, Antman EM, Braunwald E, Sabatine MS. Genetic variants in ABCB1 and CYP2C19 and cardiovascular outcomes after treatment with clopidogrel and prasugrel in the TRITON-TIMI 38 trial: a pharmacogenetic analysis. Lancet. 2010; 376:1312–19. 10.1016/S0140-6736(10)61273-1 20801494 PMC3036672

[38] Su J, Yu Q, Zhu H, Li X, Cui H, Du W, Ji L, Tong M, Zheng Y, Xu H, Zhang J, Zhu Y, Xia Y, et al. The risk of clopidogrel resistance is associated with ABCB1 polymorphisms but not promoter methylation in a Chinese Han population. PLoS One. 2017; 12:e0174511. 10.1371/journal.pone.0174511 28358842 PMC5373545

[39] Aradi D, Storey RF, Komócsi A, Trenk D, Gulba D, Kiss RG, Husted S, Bonello L, Sibbing D, Collet JP, Huber K, and Working Group on Thrombosis of the European Society of Cardiology. Expert position paper on the role of platelet function testing in patients undergoing percutaneous coronary intervention. Eur Heart J. 2014; 35:209–15. 10.1093/eurheartj/eht375 24067509 PMC3896861

[40] Zhuo ZL, Xian HP, Long Y, Liu C, Sun YY, Ma YT, Gao H, Zhao JZ, Zhao XT. Association between CYP2C19 and ABCB1 polymorphisms and clopidogrel resistance in clopidogrel-treated Chinese patients. Anatol J Cardiol. 2018; 19:123–29. 10.14744/AnatolJCardiol.2017.8097 29350207 PMC5864806

[41] Crist RC, Clarke TK, Ang A, Ambrose-Lanci LM, Lohoff FW, Saxon AJ, Ling W, Hillhouse MP, Bruce RD, Woody G, Berrettini WH. An intronic variant in OPRD1 predicts treatment outcome for opioid dependence in African-Americans. Neuropsychopharmacology. 2013; 38:2003–10. 10.1038/npp.2013.99 23612435 PMC3746708

[42] Cartegni L, Wang J, Zhu Z, Zhang MQ, Krainer AR. ESEfinder: a web resource to identify exonic splicing enhancers. Nucleic Acids Res. 2003; 31:3568–71. 10.1093/nar/gkg616 12824367 PMC169022

[43] Esmaeili R, Abdoli N, Yadegari F, Neishaboury M, Farahmand L, Kaviani A, Majidzadeh-A K. Unique CD44 intronic SNP is associated with tumor grade in breast cancer: a case control study and in silico analysis. Cancer Cell Int. 2018; 18:28. 10.1186/s12935-018-0522-2 29483847 PMC5824488

[44] Bliden KP, DiChiara J, Tantry US, Bassi AK, Chaganti SK, Gurbel PA. Increased risk in patients with high platelet aggregation receiving chronic clopidogrel therapy undergoing percutaneous coronary intervention: is the current antiplatelet therapy adequate? J Am Coll Cardiol. 2007; 49:657–66. 10.1016/j.jacc.2006.10.050 17291930

[45] Razi B, Anani Sarab G, Omidkhoda A, Alizadeh S. Multidrug resistance 1 (MDR1/ABCB1) gene polymorphism (rs1045642 C > T) and susceptibility to multiple myeloma: a systematic review and meta-analysis. Hematology. 2018; 23:456–62. 10.1080/10245332.2018.1443897 29495954

[46] Hung CC, Chiou MH, Teng YN, Hsieh YW, Huang CL, Lane HY. Functional impact of ABCB1 variants on interactions between p-glycoprotein and methadone. PLoS One. 2013; 8:e59419. 10.1371/journal.pone.0059419 23527191 PMC3602015

